# Prevalence of prediabetes and undiagnosed diabetes in a large urban middle-aged population: the CARVAR 92 cohort

**DOI:** 10.1186/s12933-023-01761-3

**Published:** 2023-02-13

**Authors:** Marie Hauguel-Moreau, Hélène Hergault, Laure Cazabat, Marion Pépin, Alain Beauchet, Vincent Aïdan, Mounir Ouadahi, Loïc Josseran, Mirella Hage, Christophe Rodon, Olivier Dubourg, Ziad Massy, Nicolas Mansencal

**Affiliations:** 1 Department of Cardiology, Ambroise Paré Hospital, Assistance Publique-Hôpitaux de Paris (AP-HP), Centre de Référence Des Cardiomyopathies Et Des Troubles du Rythme Cardiaque Héréditaires Ou Rares, Université de Versailles-Saint Quentin (UVSQ), ACTION Study Group, Paris, 9, Avenue Charles de Gaulle, 92100 Boulogne-Billancourt, France; 2 INSERM U-1018, CESP, Épidémiologie clinique, UVSQ, Villejuif, France; 3 Department of Endocrinology, Diabetology and Nutrition, Ambroise Paré Hospital, AP-HP, UVSQ, Boulogne-Billancourt, France; 4 Department of Geriatrics, Ambroise Paré Hospital, AP-HP, UVSQ, Boulogne-Billancourt, France; 5 Public Health Department, APHP, UVSQ, Boulogne-Billancourt, France; 6 Département Hospitalier d’Epidémiologie et de Santé Publique, AP-HP, GHU Paris Saclay, Hôpital Raymond-Poincaré, Garches, France; 7 Local Health insurance, Hauts de Seine Department, Paris, France; 8 Department of Nephrology, Ambroise Paré Hospital, AP-HP, UVSQ, Boulogne-Billancourt, France

**Keywords:** Prediabetes, Unknown diabetes, Prevalence, Primary prevention

## Abstract

**Background:**

The aim of this study was to assess the prevalence of prediabetes and unknown diabetes and its long-term change in a large middle-aged urban population.

**Methods:**

We conducted a screening campaign between 2007 and 2018 for cardiovascular risk factors in the western suburbs of Paris including subjects aged 40–70 (CARVAR 92). Among subjects who reported no previous diabetes, prediabetes and undiagnosed diabetes were defined as follows: fasting plasma glucose (FPG) ≥ 6.1 mmol/l (110 mg/dl) and < 7 mmol/l (126 mg/dl) for prediabetes according to WHO criteria (FPG between 5.6 and 6.9 mmol/l according to ADA criteria) and FPG ≥ 7.0 mmol/l for undiagnosed diabetes.

**Results:**

Of the 32,721 subjects in the CARVAR 92 cohort, 32,675 were included in this analysis. The median age of the patients was 56 years [30, 94], 45.4% were male, 5.9% had known diabetes, 36.4% were overweight and 18.7% obese. Among patients without previously known diabetes (n = 30,759), 8.1% had prediabetes according to WHO criteria (27.2% according to ADA criteria) and 2.3% had diabetes. Subjects with prediabetes and unknown diabetes were more likely to be male, older, and overweight or obese than non-diabetic subjects. From 2007 to 2018, the prevalence of prediabetes, unknown diabetes, and known diabetes decreased, except for prediabetes which remained stable for people aged 55–64.

**Conclusion:**

The prevalence of prediabetes and unknown diabetes remains high but decreased during a 12-year period. About one-quarter of diabetes cases remain undiagnosed. Our results highlight that there is still a room for screening and cardiovascular prevention campaigns.

*Trial registration*: IRB00012437.

**Supplementary Information:**

The online version contains supplementary material available at 10.1186/s12933-023-01761-3.

## Introduction

Diabetes mellitus is a major public health issue with a global prevalence estimated at 9.3% (537 million people) in 2021 and expected to rise in the coming decades [1]. In France, about 3.5 million people (5.3% of the French population) were treated for diabetes in 2020 [2]. While the prevalence of treated diabetes is widely studied through national health databases, studies on the prevalence of prediabetes and undiagnosed diabetes are scarce [1, 3]. However, identifying prediabetes and undiagnosed diabetes in a population is of great importance in guiding public health policy and anticipating the need for primary and secondary prevention.

The CARVAR (CARdioVAscular Risk factors) 92 study is a cardiovascular risk factors screening program conducted by our university cardiovascular department jointly with the local health insurance body since 2007 [4–8]. The aims of the present study were (1) to assess the prevalence of prediabetes and undiagnosed diabetes, and (2) to assess its long-term change in a large middle-aged urban population.

## Methods

### Study population

Between January 2007 and December 2018, we conducted a cardiovascular risk factor screening campaign in the western suburbs of Paris (the CARVAR 92 study). The target population was subjects without known cardiovascular disease (CVD) and aged between 40 and 70 years. Inhabitants of the western suburbs of Paris covered by social health insurance were sent a form inviting them to a free medical visit in one of the 17 participating centers. The following inquiries were systematically obtained: personal and family history of CVD, current cigarette smoking, and treatment with any medication. A medical examination was performed. Screening included blood tests for fast plasma glucose (FPG), total cholesterol, low-density lipoprotein-cholesterol (LDL-c), high-density lipoprotein-cholesterol (HDL-c), and triglycerides after 12 h of fasting prior to the blood draw using standardized methods. All cardiovascular risk factors were assessed. A medical report was given to the participants and sent to their general practitioners. Educational and information purposes were systematically delivered. An interview with a nutritionist and a smoking cessation specialist were offered to all study participants. The study was approved by the French Data Protection Authority (CNIL-France) and the Institutional Data Protection Authority of Foch Hospital (IRB00012437). All patients gave written informed consent.

### Definitions of prediabetes and undiagnosed diabetes

Among subjects who reported no previous diabetes mellitus, prediabetes and undiagnosed diabetes were defined according to World Health Organization (WHO) criteria [9]: 6.1 mmol/l ≤ FPG < 7 mmol/l for prediabetes and FPG ≥ 7.0 mmol/l for undiagnosed diabetes. We also studied the prevalence of prediabetes according to the American Diabetes Association (ADA) criteria [7]: 5.6 mmol/l ≤ FPG < 7 mmol/l. Glycated hemoglobin was not used in the criteria for diagnosing diabetes or prediabetes.

### Cardiovascular risk factors and 10-year risk for CVD

Known diabetes mellitus was defined as patients with a diagnosis of diabetes (treated or not) performed before the screening campaign [10]. Hypertension was defined as patients treated for hypertension or blood pressure exceeding 140 over 90 mmHg in nondiabetics and 130 over 80 mmHg in diabetic patients [11]. Dyslipidemia was defined as patients treated for dyslipidemia or high LDL-c as a fasting plasma value ≥ 4.1 mmol/L [12]. Normal weight was defined as a body mass index (BMI) < 25 kg/m^2^, overweight was defined as a BMI between 25 and 29.9 kg/m^2^, and obesity was defined as a BMI ≥ 30 kg/m^2^ [13]. Current cigarette smoking was defined as one cigarette per day for at least 6 months over the last three years. Subjects who had stopped smoking for at least 3 years were considered non-smokers.

Ten-year risk for CVD was also calculated using two scores: the modified Framingham score according to d’Agostino et al. [14] for the assessment of non-fatal and fatal CVD, and the European Systematic COronary Risk Evaluation (SCORE) for the assessment of fatal CVD [15].

### Statistical analysis

Quantitative data are expressed as mean ± standard deviation and qualitative data as frequency and percent. Analysis of variance and χ^2^ tests were used for comparisons of characteristics between those with “no diabetes”, “prediabetes” or “undiagnosed diabetes”. Linear trends were verified using the Cochran-Armitage trend test for linearity for prediabetes, unknown diabetes and known diabetes. A p value less than 0.05 was considered statistically significant. All statistical analyses were performed with R Development Core Team (2019) (R: A language and environment for statistical computing. R Foundation for Statistical Computing, Vienna, Austria).

## Results

Between January 2007 and December 2018, 32,721 subjects were included prospectively in the CARVAR 92 screening campaign. Forty-six (0.14%) were excluded for missing data regarding previous diabetes mellitus or FPG. Finally, 32,675 subjects were included with the following characteristics: median age 56 years [min 30, max 94], 45.4% male, 5.9% (95% confidence interval [CI]: 5.6–6.1%) with known diabetes, 36.4% (95% CI: 35.9–36.9%) overweight, and 18.7% (95% CI: 18.2–19.1%) obese (Table 1).

**Table 1:** Characteristics of subjects according to their diabetic status

	All	Neither diabetes nor prediabetes	Prediabetes	Unknown diabetes	Previous diabetes
	n = 32.675	n = 27.566 (84.4%)	n = 2481 (8.1%)	n = 712 (2.3%)	n = 1916 (5.9%)
Age (years)	55.6 ± 9.3	55.0 ± 9.4	58.1 ± 8.2	57.6 ± 8.2	59.9 ± 7.8
Male	14.819 (45.4%)	12.069 (43.8%)	1374 (55.4%)	448 (62.9%)	928 (48.4%)
Body mass index (kg/m^2^)	26.0 ± 4.6	25.5 ± 4.4	28.0 ± 4.8	29.5 ± 5.2	29.3 ± 5.4
Previous CVD	3595 (11.0%)	2431 (8.8%)	366 (4.8%)	110 (15.4%)	688 (35.9%)
Family history of CVD	9352 (28.6%)	8029 (29.3%)	626 (25.5%)	195 (27.7%)	502 (26.2%)
Obesity	6094 (18.7%)	4245 (15.4%)	761 (30.7%)	300 (42.1%)	788 (41.1%)
Dyslipidemia	10.505 (32.1%)	8253 (30.0%)	1006 (40.7%)	301 (42.5%)	945 (49.3%)
Hypertension	10.539 (32.2%)	7925 (28.8%)	1168 (47.1%)	407 (57.2%)	1039 (54.2%)
Current smoking	5999 (18.3%)	5173 (18.8%)	402 (16.2%)	144 (20.2%)	280 (14.6%)
Systolic BP (mmHg)	125.9 ± 15.5	125.0 ± 14.9	131.4 ± 16.7	136.7 ± 18.5	132.9 ± 16.8
Diastolic BP (mmHg)	76.8 ± 9.7	76.4 ± 9.5	79.2 ± 10.2	81.7 ± 11.1	78.5 ± 9.9
Fasting glycemia (g/L)	0.99 ± 0.21	0.93 ± 0.09	1.16 ± 0.04	1.50 ± 0.37	1.39 ± 0.47
Fasting total cholesterol (g/L)	2.15 ± 0.39	2.16 ± 0.38	2.18 ± 0.40	2.21 ± 0.45	1.90 ± 0.41
Triglycerides (g/l)	1.12 ± 0.66	1.06 ± 0.61	1.35 ± 0.81	1.58 ± 0.91	1.40 ± 0.86
Fasting HDLc (g/L)	0.60 ± 0.18	0.61 ± 0.18	0.55 ± 0.15	0.51 ± 0.15	0.52 ± 0.19
Fasting LDLc (g/L)	1.33 ± 0.34	1.34 ± 0.34	1.37 ± 0.36	1.39 ± 0.40	1.11 ± 0.35
10-year risk for CVD (d’Agostino et al.) (%)	11.4 ± 9.8	9.7 ± 7.7	14.4 ± 9.7	28.8 ± 16.8	25.1 ± 14.6
10-year risk of fatal CVD (SCORE) (%)	1.87 ± 2.16	1.72 (2.03)	2.54 (2.47)	NA	NA

*BP* blood pressure, *CVD* cardiovascular disease, *HDLc* high-density lipoprotein cholesterol, *LDLc* low-density lipoprotein cholesterol

**Fig. 1:**
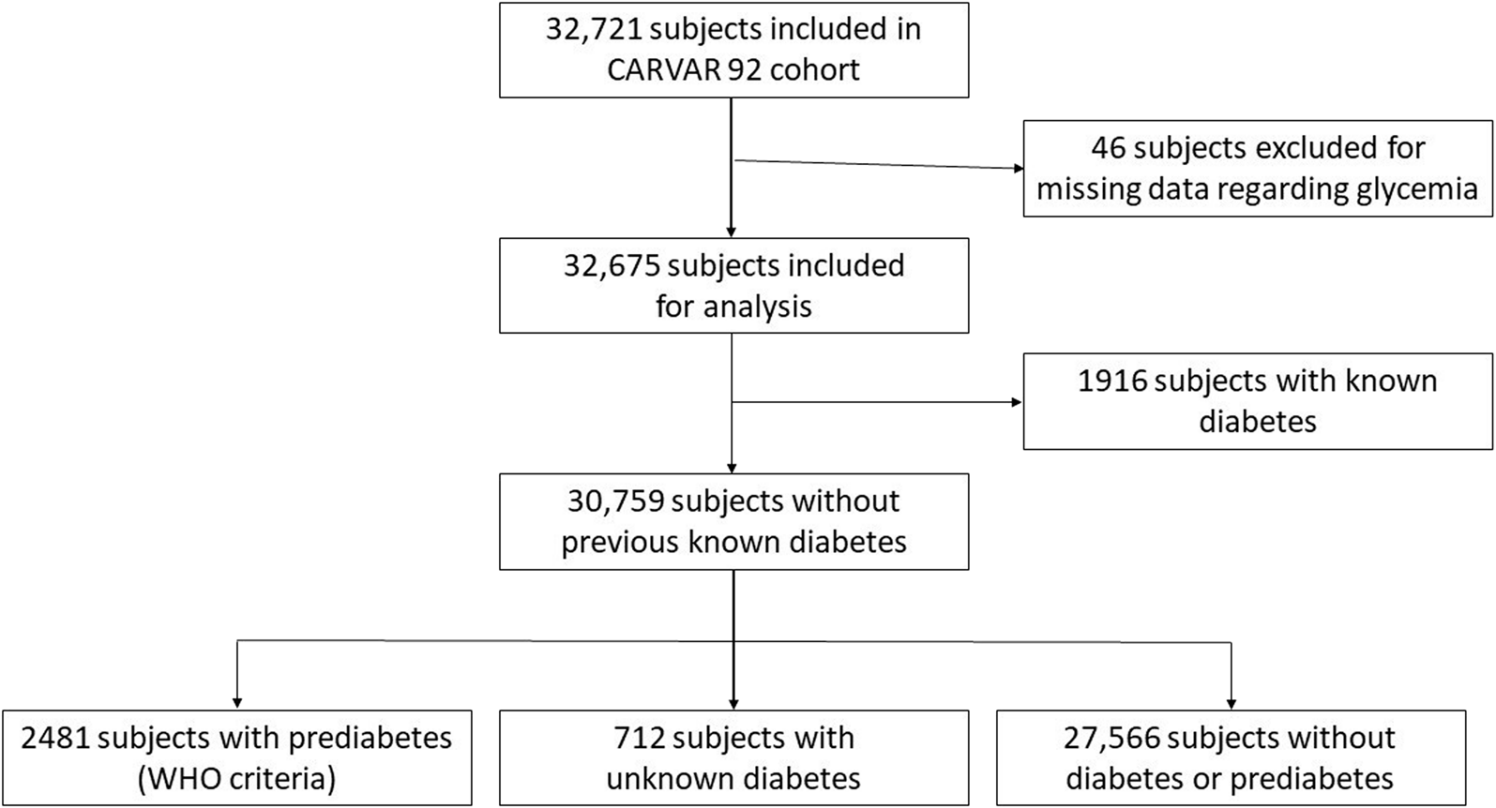
Study flow chart

Among patients without previous known diabetes (n = 30,759) and according to WHO criteria, 2481 subjects (8.1%, 95% CI 7.8–8.4%) had prediabetes and 712 (2.3%, 95% CI 2.1–2.5%) had diabetes (Fig. 1). The characteristics of subjects with prediabetes according to WHO criteria were as follows: 55.4% male, mean age 58.1 ± 8.2 years-old, mean BMI 28 ± 4.8 kg/m^2^, 30.7% with obesity and 47.1% with hypertension. According to ADA criteria, 8359 subjects (27.2%, 95% CI 26.7–27.7%) presented with prediabetes (Additional file 1: Table S1). As compared to subjects with prediabetes according to WHO criteria, subjects with prediabetes according to ADA criteria were more likely to be younger, had lower BMI, and had less hypertension and dyslipidemia. Among subjects with diabetes (n = 2628), 27% had unknown diabetes. Subjects with unknown diabetes were more likely to be male as compared to non-diabetic subjects (62.9% versus 43.8%, p < 0.01), were more frequently obese (42.1% versus 15.4%, p < 0.001), had dyslipidemia (42.5% versus 30.0%, p < 0.01), and had hypertension (57.2% versus 28.8%, p < 0.001) (Table 1). Ten-year risk for CVD was significantly higher among patients with prediabetes as compared to non-diabetic patients, and among patients with unknown diabetes or known diabetes as compared to patients with prediabetes and to non-diabetic patients. Ten-year risk for fatal CVD was significantly higher among patients with prediabetes as compared to non-diabetic patients.

Table 2 presents the prevalence of prediabetes, unknown diabetes, previous diabetes, and total diabetes (unknown diabetes and previous diabetes) according to age and sex. The prevalence of prediabetes and unknown diabetes doubled between subjects aged < 50 years and subjects ≥ 50 years. Ten percent (95% CI 9.5–10.6) of subjects over 60 years old presented with prediabetes. Total diabetes prevalence increased with age, reaching 11.1% (95% CI 10.1–12.1) in subjects over 60 years old. In all age categories, prevalence of prediabetes, unknown diabetes, previous diabetes, and total diabetes were higher in men than in women (Table 2).

**Table 2:** Weighted prevalence of prediabetes, unknown diabetes and previous diabetes according to age and sex

	Age < 50 years old			50 ≤ Age < 60 years old			Age ≥ 60 years old		
	Total	Men	Women	Total	Men	Women	Total	Men	Women
n = 32,675	n = 8994	n = 5234	n = 3740	n = 11,787	n = 5659	n = 6128	n = 11,894	n = 3923	n = 7971
Prediabetes	4.3 (3.9–4.7)	5.3 (4.7–6.0)	2.9 (2.4–3.5)	9.1 (8.5–9.6)	12.0 (11.1–12.9)	6.4 (5.8–7.1)	10.0 (9.5–10.6)	13.3 (12.4–14.5)	8.5 (7.8–9.1)
Unknown diabetes	1.4 (1.2–1.7)	1.6 (1.3–2.0)	1.1 (0.8–1.5)	2.7 (2.4–3.1)	3.9 (3.4–4.5)	1.7 (1.3–2.0)	2.6 (2.3–2.9)	4.5 (3.8–5.3)	1.7 (1.4–2.0)
Previous diabetes	2.0 (1.7–2.3)	2.0 (1.5–2.5)	2.0 (1.6–2.4)	6.2 (5.8–6.7)	7.1 (6.4–7.8)	5.4 (5.1–6.3)	8.5 (8.0–9.0)	14.4 (13.3–15.5)	5.6 (5.1–6.1)
Total diabetes	3.4 (3.0–3.8)	3.6 (3.0–4.2)	3.1 (2.6–3.6)	8.9 (8.3–9.4)	11.0 (9.9–12.1)	7.1 (6.6–7.6)	11.1 (10.1–12.1)	18.9 (17.0–20.8)	7.3 (6.8–7.8)

Data are presented as percent (95% confidence interval)

Table 3 presents the prevalence of prediabetes, unknown diabetes, previous diabetes, and total diabetes according to BMI classes (normal weight, overweight, obesity) and sex. Prediabetes, unknown diabetes, previous diabetes, and total diabetes were significantly more frequent in subjects with overweight than normal weight and in subjects with obesity than subjects with overweight. In subjects with obesity, prevalence of prediabetes reached 14.3% (95% CI 13.4–15.3), whereas unknown diabetes prevalence was 5.7% (95% CI 5.0–6.3).

**Table 3:** Weighted prevalence of prediabetes, unknown diabetes and previous diabetes according to body mass index and sex

	Normal weight BMI < 25 kg/m^2^			Overweight 25 ≤ BMI < 30 kg/m^2^			Obesity BMI ≥ 30 kg/m^2^		
	Total	Men	Women	Total	Men	Women	Total	Men	Women
n = 32,675	n = 14,692	n = 5822	n = 8870	n = 11,887	n = 6635	n = 5252	n = 6096	n = 2354	n = 3742
Prediabetes	4.5 (4.2–4.9)	6.4 (5.8–7.1)	3.3 (3.0–3.7)	9.6 (9.1–10.2)	10.9 (10.1–11.7)	8.0 (7.3–8.8)	14.3 (13.4–15.3)	16.3 (14.8–18.0)	13.1 (11.9–14.3)
Unknown diabetes	0.8 (0.7–1.0)	1.2 (0.9–1.5)	0.5 (0.4–0.7)	2.7 (2.4–3.0)	3.5 (3.0–4.0)	1.7 (1.3–2.1)	5.7 (5.0–6.3)	7.8 (6.7–9.1)	4.2 (3.6–5.0)
Previous diabetes	2.6 (2.4–2.9)	3.9 (3.4–4.4)	1.8 (1.7–1.8)	6.2 (5.8–6.7)	6.6 (6.0–7.2)	5.8 (5.2–6.5)	12.9 (12.1–13.8)	11.3 (10.0–12.6)	14.0 (12.9–15.1)
Total diabetes	3.4 (3.0–3.8)	5.1 (4.8–5.4)	2.3 (1.7–2.9)	9.9 (9.1–10.7)	10.1 (9.1–11.2)	7.5 (6.9–8.1)	18.6 (16.8–20.4)	20.1 (18.5–21.7)	18.2 (17.6–18.8)

Data are presented as percent (95% confidence interval)

*BMI* body mass index

**Fig. 2:**
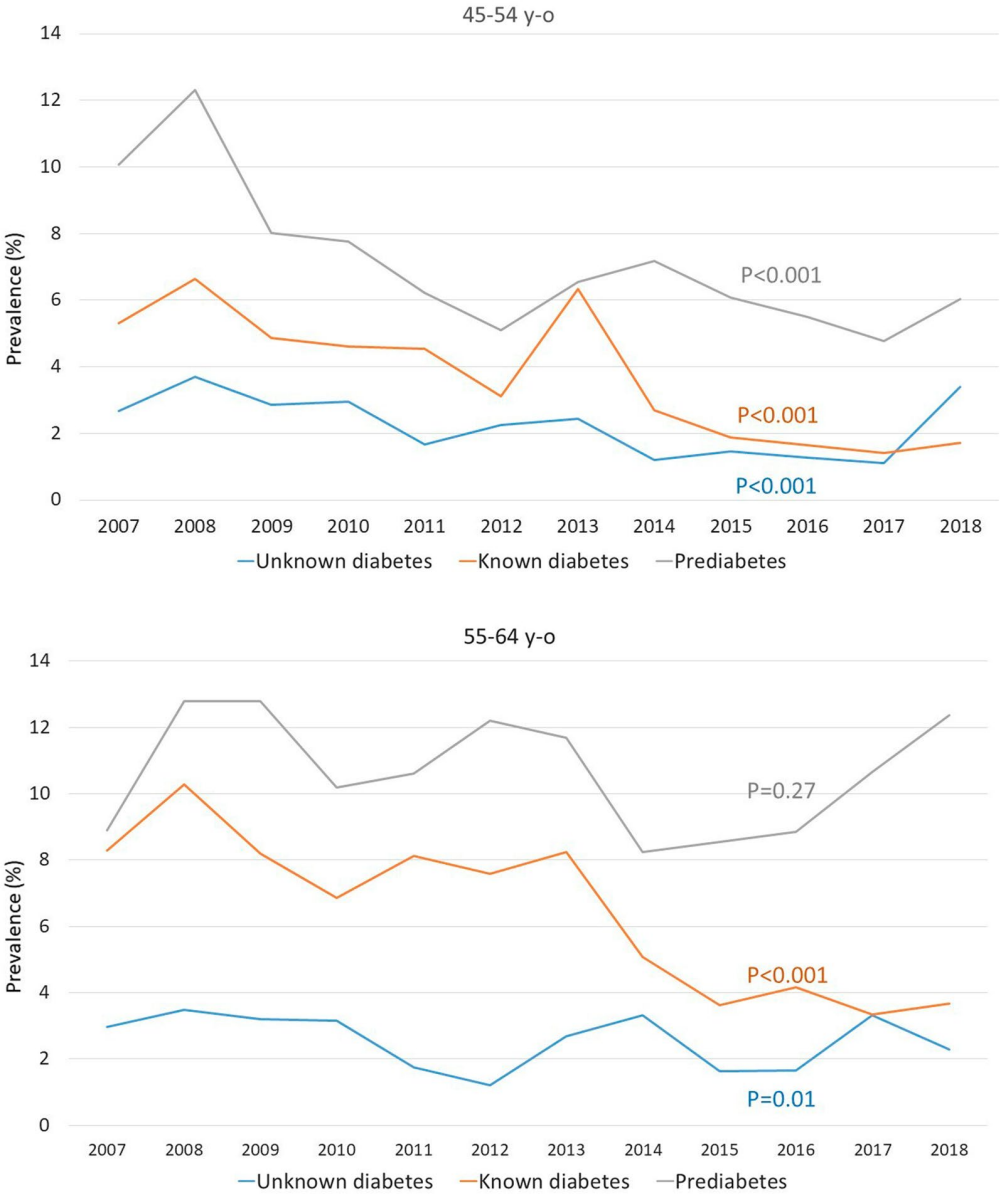
Change in the prevalence of prediabetes according to WHO criteria (grey line), known diabetes (orange line) and unknown diabetes (blue line) from 2007 to 2018 in subjects aged between 45 and 64 years old

From 2007 to 2018 (Fig. 2), the prevalence of prediabetes, unknown diabetes, and previous diabetes decreased in patients aged 45–64 years old (p for trend < 0.001 for all), except for prediabetes which remained stable in subjects aged 55–64 years old (p for trend 0.27).

## Discussion

In this large middle-aged French cohort, we observed a prevalence of 8.1% of prediabetes based on FPG alone (27.2% according to the ADA definition), 2.3% of unknown diabetes and 5.9% of previous diabetes. All these rates were higher in men, increased steadily with age and BMI, but decreased during a 12-year period, except for prediabetes which remained stable in subjects aged 55–64.

Prediabetes is considered as a high-risk state for diabetes development and needs to be considered in order to avoid excess cardiovascular morbidity [16]: 5–10% of people per year with prediabetes will progress to diabetes. Observational evidence shows associations between prediabetes and early forms of micro- and macrovascular disease [17]. In our study, 10-year risk for fatal and non-fatal CVD were significantly higher in patients with prediabetes as compared to non-diabetic patients. Several studies have evaluated the prevalence of prediabetes worldwide: 9.9% and 11% according to WHO criteria in the ESTEBAN French survey [3] and in an English national cohort [18], and 25% and 23.9% according to ADA criteria in Luxembourg [19], and in South Korea [20]. These results are consistent with our results: 8.1% and 27.2% according to WHO and ADA criteria, respectively. However, this prevalence was significantly lower, as compared to the prevalence observed in the USA (38% according to ADA criteria) [21]. The characteristics of the European and American populations may explain in part these differences concerning the prevalence of prediabetes. For prediabetic individuals, lifestyle modification is the cornerstone of diabetes prevention, with evidence of a 40–70% reduction in relative risk of diabetes. In our study, we found a clear association between prediabetes and overweight or obesity.

Screening campaigns can detect unknown disease in a population: 2.3% of our cohort presented with unknown diabetes which is consistent with recent European cohorts (1.6–1.7%) [3, 18, 19]. In other words, among subjects with diabetes, 27% were unaware of their condition. Screening for abnormal glucose metabolism, especially in men after 50 years of age, particularly if overweight or obese, is the first step in identifying the disorder and initiating lifestyle modification. Indeed, earlier diagnosis and treatment of diabetes leads to a reduction in mortality rates among patients with diabetes [20, 22, 23].

In the present study, the prevalence of prediabetes and unknown diabetes increased steadily with male sex, age, and BMI. Previous studies reported higher rates of men in prediabetes and diabetes subjects [19, 24], whereas a South Korean study reported no association with sex [25]. Age was also a major correlate with prediabetes and diabetes [18, 19], with a remarkable abrupt increase after age 50 in this cohort, as reported in diabetic Korean women [25]. Overweight and obesity were statistically associated with prediabetes and unknown diabetes, with a threefold increase between normal weight and obesity for prediabetes, and a sevenfold increase for unknown diabetes as already demonstrated in previous studies [18, 19, 25]. Our study provides an "identikit picture" of the unmissable prediabetes and diabetes screening subject: man > 50 years old and BMI > 25 kg/m^2^. However, prediabetes and diabetes screening should not be forgotten in women, as prediabetes and unknown diabetes rates double between women < 50 years old and women > 50 years old.

In our cohort, the prevalence of prediabetes, unknown diabetes, and previous diabetes decreased in subjects aged 45–64 between 2007 and 2018, except for prediabetes which remained stable in subjects aged 55–64. One may ask if the type 2 diabetes epidemic is plateauing. In a recent French nationwide population-based study, type 2 diabetes prevalence increased slightly between 2010 and 2017, whereas its incidence decreased [26] as well as in USA [27] and Sweden [28]. In that French study [26], prevalence rates decreased in people aged 45–65 years, which is the target population of our screening campaign. An explanation for the decrease in prediabetes and unknown diabetes would be the stability of obesity prevalence in France, as previously shown in our cohort [6]. Repeated 5-year Nutrition and Health National Plans (PNNS 4) and the 2016 introduction of the Nutri-score label on food products may have limited the epidemic.

Our study has several limitations. First, CARVAR 92 is a screening program conducted in the second richest area in France, but presenting high socio-economic disparities among cities and hence is representative of the general population. However, this large clinical screening campaign (> 30,000 people) has been going on for more than 12 years, using the same protocol. All risk factors were systematically assessed, especially those strongly associated with diabetes (age, sex, BMI), whereas this is not the case in the health administrative database [3] . These relevant characteristics allow us to carry out a thorough analysis on prediabetes and unknown diabetes. Second, no data on ethnicity were available because of French legislation, whereas ethnicity is a factor strongly associated with diabetes mellitus. Third, FPG was tested only once without any glycated hemoglobin. This may underestimate the real prevalence of prediabetes and unknown diabetes. Finally, it would be useful to harmonize the definitions of prediabetes (ADA/WHO). However, we have presented both definitions to allow comparisons between studies and to inform health policies unequivocally.

## Conclusion

Our results show that the prevalence of prediabetes and unknown diabetes is high, but is decreasing in France over a 12-year period, and about one-quarter of diabetes cases remain undiagnosed. We provide an "identikit picture" of the unmissable prediabetes and diabetes screening subject: man > 50 years old and overweight. These results highlight the need to support primary prevention, and to enhance secondary prevention of prediabetes and diabetes, especially through promotion of screening in populations at risk.

## Supplementary Information

**Additional file 1: Table S1.** Characteristics of subjects with prediabetes according to the definition of prediabetes (WHO versus ADA criteria).

## Data Availability

The datasets generated and/or analysed during the current study are not publicly available due to personal data but are available from the corresponding author on reasonable request.
