# Supplementary material for: Prevalence of prediabetes and undiagnosed diabetes in a large urban middle-aged population: the CARVAR 92 cohort
Source: Cardiovasc Diabetol. 2023 Feb 13;22:31. doi: 10.1186/s12933-023-01761-3 (PMC9926717; doi:10.1186/s12933-023-01761-3)
Supplement: Supplementary file 1 — Additional file 1: Table S1. Characteristics of subjects with prediabetes according to the definition of prediabetes (WHO versus ADA criteria). [file 12933_2023_1761_MOESM1_ESM.docx]

**Supplementary table 1:** Characteristics of subjects with prediabetes according to the definition of prediabetes (WHO versus ADA criteria)

|  | **Prediabetes (WHO)** | **Prediabetes (ADA)** | **p** |
| --- | --- | --- | --- |
|  | ***n=2481 (8.1%)*** | ***n=8359 (27.2%)*** |  |
| Age (years) | 58.1 ± 8.2 | 57.03± 7.9 | <0.01 |
| Male | 1374 (55.4%) | 4510 (54.0%) | 0.21 |
| Body mass index (kg/m²) | 28.0 ± 4.8 | 27.2 ± 5.2 | <0.001 |
| Previous CVD | 366 (4.8%) | 990 (11.8%) | <0.001 |
| Family history of CVD | 626 (25.5%) | 2166 (25.9%) | 0.46 |
| Obesity | 761 (30.7%) | 2065 (24.7%) | <0.001 |
| Dyslipidemia | 1006 (40.7%) | 3159 (37.8%) | 0.01 |
| Hypertension | 1168 (47.1%) | 3382 (40.5%) | <0.001 |
| Current smoking | 402 (16.2%) | 1451 (17.4%) | 0.18 |
| Systolic BP (mmHg) | 131.4 ± 16.7 | 129.0 ± 15.9 | <0.001 |
| Diastolic BP (mmHg) | 79.2 ± 10.2 | 78.5 ± 9.7 | 0.004 |
| Fasting glycemia (g/L) | 1.16 ± 0.04 | 1.08 ± 0.04 | <0.001 |
| Fasting total cholesterol (g/L) | 2.18 ± 0.40 | 2.19 ± 0.39 | 0.35 |
| Triglycerides (g/l) | 1.35 ± 0.81 | 1.24 ± 0.87 | <0.001 |
| Fasting HDLc (g/L) | 0.55 ± 0.15 | 0.57 ± 0.12 | <0.001 |
| Fasting LDLc (g/L) | 1.37 ± 0.36 | 1.38 ±0.35 | 0.16 |
| 10-year risk for CVD (d’Agostino et al.) (%) | 14.4 ± 9.7 | 12.9 ± 8.7 | <0.001 |
| 10-year risk of fatal CVD (SCORE) (%) | 2.5 ± 2.5 | 2.3 ±2.3 | <0.001 |

BP = blood pressure; CVD = cardiovascular disease; HDLc = high-density lipoprotein cholesterol; LDLc = low-density lipoprotein cholesterol.
